# Er^3+^/Yb^3+^ co-doped ZnS quantum dots: structure, optical properties and up-conversion luminescence

**DOI:** 10.1039/d6ra00395h

**Published:** 2026-03-05

**Authors:** L. T. T. Ngan, V. H. Yen, N. T. Hien, N. T. K. Van, N. V. Ha, N. T. Luyen, N. D. Vinh, N. X. Ca

**Affiliations:** a Faculty of Engineering and Technology, Thai Nguyen University of Information and Communication Technology Thai Nguyen Vietnam; b Institute of Science and Technology, TNU – University of Sciences Thai Nguyen Vietnam canx@tnus.edu.vn; c Faculty of Natural Sciences and Technology, TNU – University of Sciences Thai Nguyen Vietnam

## Abstract

Er^3+^/Yb^3+^ co-doped ZnS quantum dots (QDs) were synthesized using a wet chemical approach and studied for their structural and optical properties, as well as upconversion (UC) luminescence. X-ray diffraction (XRD) confirmed the formation of phase cubic ZnS, where rare-earth Er^3+^ and Yb^3+^ ions were effectively incorporated into the host lattice without secondary phases. X-ray photoelectron spectroscopy (XPS) analyses further verified the trivalent states of Er and Yb ions in the QDs. For the first time, the UC luminescence phenomenon in Er^3+^/Yb^3+^ co-doped ZnS QDs was studied and explained in detail. Under 980 nm excitation, the Er^3+^/Yb^3+^ co-doped ZnS QDs exhibited green and red UC bands, dominated by the green emission, whose intensity strongly depended on Yb^3+^ concentration. Power-dependent and lifetime measurements indicated that the UC process was primarily governed by a two-photon mechanism facilitated by efficient Yb^3+^ → Er^3+^ energy transfer. Chromaticity analyses demonstrated a distinct emission color shift from deep-blue (pure ZnS QDs) to stable green-yellow in co-doped QDs. These results highlight the potential of Er^3+^/Yb^3+^ co-doped ZnS QDs as efficient UC nanomaterials for applications in photonic and optoelectronic devices.

## Abstract 2

Upconversion emission spectra of ZnEr1%Yb1%S QDs under 980 nm excitation (^4^I_15/2_–^4^I_11/2_) with excitation power varying from 1 to 10 mW. The inset shows the dependence of the upconversion emission intensity on the excitation power.

## Graphical abstract

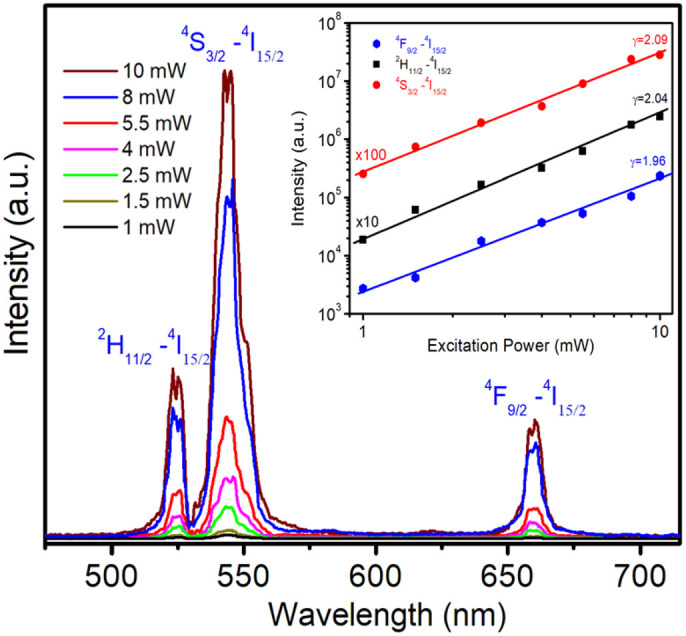

## 1. Introduction

Upconversion (UC) luminescent materials have garnered significant interest due to their ability to convert low-energy near-infrared (NIR) radiation into higher-energy visible or ultraviolet emissions through a nonlinear optical process.^1–3^ This phenomenon, which involves sequential absorption of two or more NIR photons and subsequent emission of a shorter-wavelength photon, is particularly advantageous for applications in photovoltaics, optical sensing, bioimaging, anti-counterfeiting, and solid-state lighting.^4,5^ Among the various host materials investigated for upconversion processes, II–VI semiconductors such as ZnO,^6^ ZnSe,^7^ and ZnS^8^ are of notable interest owing to their wide bandgap, high exciton binding energy, and favorable chemical stability. Zinc sulfide (ZnS), in particular, with a direct bandgap of approximately 3.6 eV, offers high optical transparency in the visible region and low phonon energy, which reduces non-radiative losses and enhances radiative transitions. Moreover, ZnS is chemically more stable under reducing conditions compared to ZnO or ZnSe and provides good lattice compatibility for rare-earth ion incorporation.^9^

Trivalent lanthanide ions, especially erbium (Er^3+^) and ytterbium (Yb^3+^), are extensively employed as activator–sensitizer pairs in upconversion systems.^10,11^ Yb^3+^ ions exhibit a simple two-level energy structure with a strong absorption band around 980 nm corresponding to the ^2^F_7/2_ → ^2^F_5/2_ transition, making them efficient sensitizers.^12^ In contrast, Er^3+^ ions possess multiple metastable states, such as ^4^I_11/2_, ^4^F_7/2_, ^4^S_3/2_, and ^4^F_9/2_, which enable emission in the green (∼540 nm) and red (∼660 nm) regions.^13^ Upon energy transfer from excited Yb^3+^ ions, Er^3+^ ions can be sequentially excited to higher energy levels and emit visible light through radiative decay. This energy transfer upconversion (ETU) process is typically more efficient than excited-state absorption (ESA) in isolated Er^3+^ systems due to the higher absorption cross-section of Yb^3+^.^14,15^

Upconversion luminescence based on the cooperative mechanism between rare-earth ions such as Er^3+^ and Yb^3+^ has been effectively applied in various host systems, particularly in fluoride and oxide-based materials such as NaYF_4_,^16^ Y_2_O_3_,^17^ and TiO_2_.^18^ These hosts are favored due to their high emission efficiency and low reabsorption losses. However, their relatively large particle sizes often limit their suitability for biomedical and *in vivo* bio-labeling applications. In contrast, zinc sulfide quantum dots (ZnS QDs), with characteristic sizes of just a few nanometers, exhibit several advantages such as non-toxicity, strong photoluminescence, excellent stability, and good dispersibility in aqueous media. As a wide-bandgap semiconductor, ZnS allows for easy tuning of optical properties through quantum confinement effects. Previous studies have highlighted the potential of ZnS QDs in applications such as bio-labeling, sensing, and display technologies.^19–21^ Nevertheless, most of these works have focused on conventional photoluminescence or size-dependent emission behavior, while the upconversion luminescence (UC) properties remain largely unexplored.

Notably, to the best of our knowledge, no studies have yet reported the upconversion luminescence behavior of ZnS quantum dots co-doped with Er^3+^ and Yb^3+^ ions. Incorporating these two rare-earth ions into a strongly quantum-confined host such as ZnS QDs presents considerable challenges but also offers promising opportunities for studying nonlinear absorption, energy transfer, and emission mechanisms. The efficiency of the UC process in such systems has not been fully clarified, particularly with respect to influencing factors such as dopant concentration, quantum dot size, surface states, and synthesis conditions. In this work, we report the synthesis of Er^3+^/Yb^3+^ co-doped ZnS QDs *via* a wet chemical method and systematically investigate their structural, optical, and upconversion luminescence properties. Special attention is paid to the role of rare earth ion concentration and the nature of the UC luminescence process is explained in detail. Our findings provide insights into the design of efficient ZnS-based UC materials for next-generation photonic and optoelectronic devices.

## 2. Experimental

### 2.1. Materials

Zinc acetate dihydrate [Zn(CH_3_COO)_2_·2H_2_O, ≥99%], erbium(iii) chloride hexahydrate [ErCl_3_·6H_2_O, 99.9%], ytterbium(iii) chloride hexahydrate [YbCl_3_·6H_2_O, 99.9%], sulfur powder (S, ≥99.5%), oleic acid (OA, 90%), 1-octadecene (ODE, 90%), and trioctylphosphine (TOP, 90%) were purchased from Sigma-Aldrich and used without further purification.

### 2.2. Synthesis of Er^3+^/Yb^3+^ co-doped ZnS quantum dots

The Er^3+^/Yb^3+^ co-doped ZnS QDs were synthesized *via* a wet chemical route in an ODE solvent as the reaction medium. The synthesis procedure was conducted under an inert atmosphere using standard Schlenk line techniques. In a typical synthesis, Zn(CH_3_COO)_2_·2H_2_O, ErCl_3_·6H_2_O, and YbCl_3_·6H_2_O were added into a three-necked flask containing 10 mL of ODE and 2 mL of OA. The mixture was magnetically stirred under vacuum at 120 °C for 30 minutes to remove residual water and oxygen. After degassing, the system is placed in a nitrogen atmosphere and the temperature is increased to 180 °C. Separately, a sulfur precursor solution was prepared by dissolving 0.5 mmol of elemental sulfur in 2 mL of TOP under mild heating (∼60 °C). Once a transparent yellow solution was obtained, it was swiftly injected into the hot metal precursor solution at 180 °C. Immediately after injection, the temperature was raised to 220 °C and maintained for 60 minutes to allow crystal growth. After completion, the reaction mixture was cooled to room temperature. The Er^3+^/Yb^3+^ co-doped ZnS QDs were precipitated by adding excess ethanol, followed by centrifugation at 8000 rpm for 10 minutes. The solution containing the QDs was mixed with ethanol and centrifuged twice to remove unreacted precursors and excess ligands. Finally, the purified QDs were redispersed in hexane for storage and characterization.

### 2.3. Characterization methods

The crystal structure of the Er^3+^/Yb^3+^ co-doped ZnS QDs was identified using X-ray diffraction (XRD, Bruker D8 Advance, Cu Kα radiation, *λ* = 1.5406 Å). Ultraviolet-visible (UV-vis) absorption spectra of the NCs were recorded using a Jasco V-770 spectrometer (Varian). Photoluminescence (PL), upconversion PL, photoluminescence excitation (PLE) spectra, and decay time curves were measured using an FLS1000 spectrophotometric system equipped with a 450 W Xe lamp. X-ray photoelectron spectroscopy (XPS) analysis was performed on a Thermo VG Escalab 250 photoelectron spectrometer to analyze the elemental composition and chemical states. The particle size and morphology were characterized by transmission electron microscopy (TEM, JEOL JEM-1010) operated at an accelerating voltage of 80 kV.

## 3. Results and discussion

### 3.1. Structural analysis

**Fig. 1:**
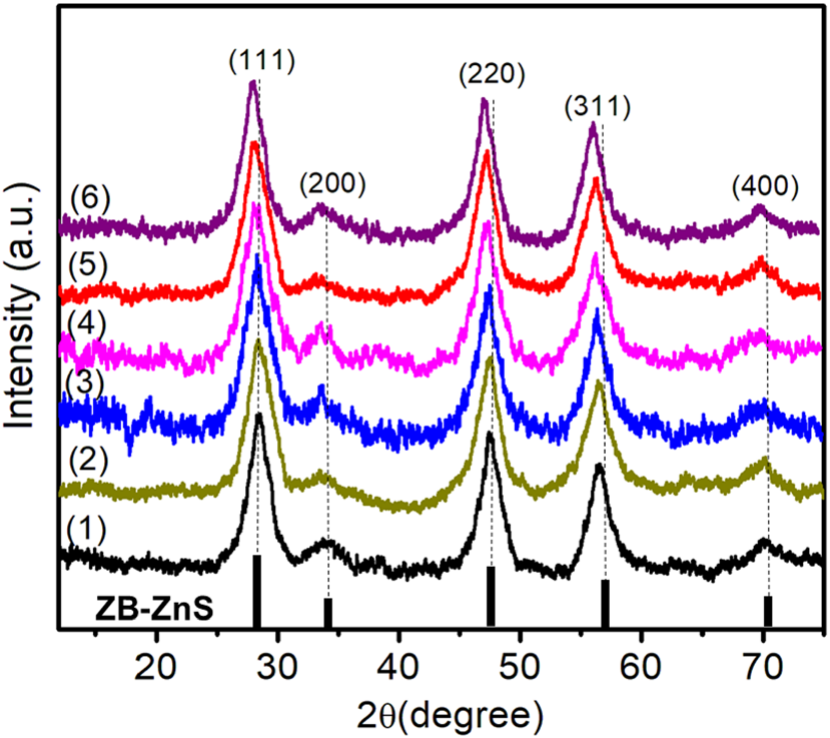
XRD pattern of QDs: (1) ZnS, (2) ZnEr1%Yb0.5%S, (3) ZnEr1%Yb1%S, (4) ZnEr1%Yb2%S, ZnEr1%Yb5%S, and (6) Zn1%Er10%YbS.

The crystallographic structure of the synthesized pure ZnS and Er^3+^/Yb^3+^ co-doped ZnS QDs were examined by X-ray diffraction (XRD), and the corresponding patterns are presented in Fig. 1. All samples exhibited a consistent set of diffraction peaks located at 2*θ* values around 28.5°, 33.0°, 47.5°, 56.3°, and 69.5°, which are indexed to the (111), (200), (220), (311), and (400) planes, respectively. These diffraction peaks are in excellent agreement with the standard cubic zinc blende (ZB) structure of ZnS (JCPDS card no. 80-0020), confirming the formation of single-phase cubic ZnS in all cases.^22^ The absence of any secondary phases or impurity-related peaks indicates that both Er and Yb dopants were successfully incorporated into the ZnS host lattice without forming separate oxide or sulfide phases such as Er_2_O_3_ or Yb_2_O_3_. This suggests high chemical compatibility and effective substitution or interstitial doping of Er^3+^ and Yb^3+^ ions into the ZnS lattice. Moreover, the consistent peak positions among all samples further confirm that the doping process did not significantly alter the crystal symmetry or induce any phase transformation.

**Fig. 2:**
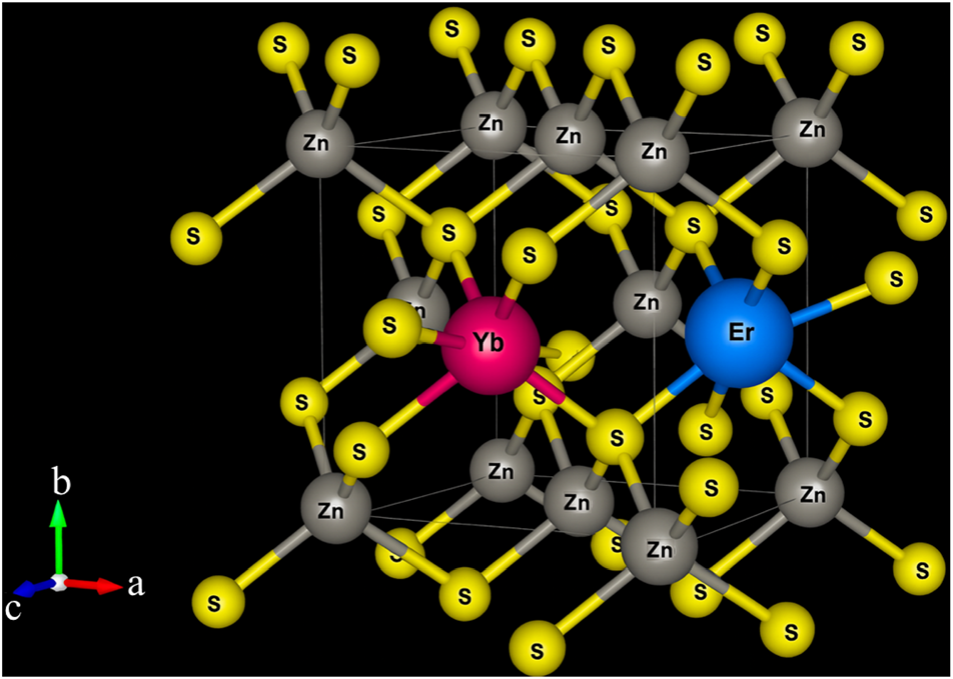
The unit-cells scheme of Er^3+^/Yb^3+^ co-doped ZnS QDs.

The strong intensity of the (111) reflection compared to other planes suggests a preferential growth orientation along the (111) direction, which is characteristic of the ZB phase of ZnS. It is well known that rare-earth ions can influence nucleation and crystal growth behavior through their high charge and ionic radius, which can interact with the sulfur or zinc sublattices, affecting energy barriers for grain boundary migration.^23^ A slight shift of the diffraction peaks toward lower 2*θ* angles is observed with increasing concentrations of Er and Yb dopants, indicating a progressive expansion of the ZnS lattice. This behavior can be explained by Bragg's law, where a decrease in the diffraction angle corresponds to an increase in the interplanar spacing (*d*), suggesting an enlargement of the unit cell. The lattice expansion is likely attributed to the partial substitution of Zn^2+^ ions (ionic radius ≈ 0.74 Å) by the larger Er^3+^ (≈0.89 Å) and Yb^3+^ (≈0.868 Å) ions.^24,25^ The unit cell illustration with the cubic structure of Er^3+^/Yb^3+^ co-doped ZnS QDs is shown in Fig. 2. The crystallite size (*D*) was determined from the broadening of the strongest peak (111) in the XRD pattern using the Debye–Scherrer equation:^13,26^1
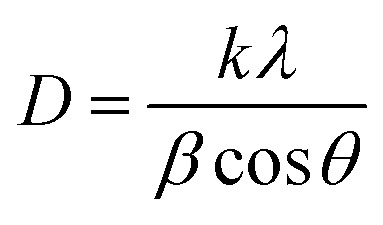
Here, *λ* is the X-ray wavelength, *β* is the full width of the diffraction line at half its maximum intensity, and *θ* is the Bragg angle.

The crystal lattice constant (*a*) was obtained from the XRD data using the following equation:^21,27^2
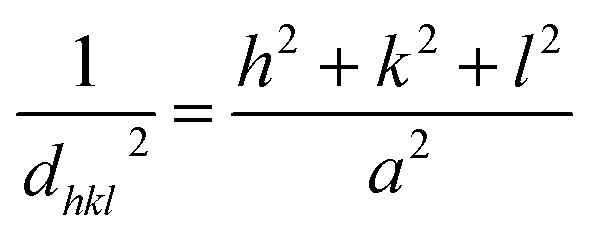
*d* represents the distance between the lattice planes corresponding to Miller indices *h*, *k*, *l* and is determined using Bragg's equation:^21,27^

3 *nλ* = 2*d*_*hkl*_ sin *θ*

Despite the low dopant concentrations, the incorporation of these larger ions induces localized lattice distortions, which cumulatively result in a measurable increase in lattice parameters. In addition, the replacement of divalent Zn^2+^ by trivalent Er^3+^ and Yb^3+^ may introduce a charge imbalance in the crystal lattice. This imbalance is commonly compensated by the generation of cation vacancies or interstitial defects, further contributing to the lattice expansion. Such a mechanism is consistent with previous reports on II–VI semiconductor systems doped with rare-earth ions, and it confirms the successful incorporation of RE^3+^ species into the ZnS host without the formation of secondary impurity phases. The crystallite strain (*ε*) in the QDs can be estimated from the peak broadening using Williamson–Hall equation:^28^4
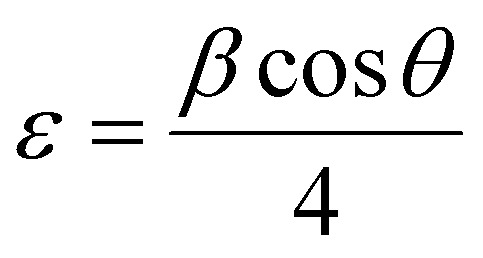


The parameters were calculated from the XRD data, including *a*, 2*θ*, *β*, *d*_*hkl*_, *D*, and *ε* are presented in Table 1. The structural parameters of Er^3+^/Yb^3+^ co-doped ZnS QDs reveal systematic changes with increasing Yb^3+^ content. The (111) diffraction peak gradually shifts toward lower 2*θ* values (28.423 → 28.062°), accompanied by a increase in the lattice parameter (*a* = 5.412 → 5.483 Å) and interplanar spacing, indicating lattice expansion due to the substitution of larger Er^3+^/Yb^3+^ ions for Zn^2+^. The crystallite size (*D*) initially decreases from 3.50 to 3.02 nm at low-medium doping levels, suggesting that lattice disorder and defect formation hinder crystal growth. However, at higher Yb^3+^ concentrations, *D* increases again, implying that Yb–S bond may promote recrystallization. The microstrain (*ε*) follows an opposite trend: it rises from 9.89 × 10^−3^ to a maximum of 11.46 × 10^−3^ at 2% Yb, reflecting increased lattice distortion, then decreases slightly at higher doping, likely due to stress relaxation *via* defect redistribution or surface complex formation. Overall, the results demonstrate that rare-earth codoping induces both lattice expansion and strain modulation in ZnS QDs, strongly influencing their structural stability and crystallite evolution.

**Table 1:** Lattice parameters, micro-strain, and crystallite size of obtained QDs

Sample	2*θ* (deg.)	*β* (deg.)	*d*_*hkl*_ (nm)	*a* (Å)	*D* (nm)	*ε* × 10^−3^
ZnS	28.423	2.342	3.125	5.412	3.502	9.893
ZnEr1%Yb0.5%S	28.407	2.494	3.128	5.417	3.291	10.528
ZnEr1%Yb1%S	28.318	2.641	3.133	5.426	3.104	11.164
ZnEr1%Yb2%S	28.285	2.717	3.142	5.442	3.023	11.461
ZnEr1%Yb5%S	28.146	2.565	3.150	5.456	3.199	10.829
ZnEr1%Yb10%S	28.062	2.482	3.166	5.483	3.302	10.493

### 3.2. Elemental and chemical composition analysis

**Fig. 3:**
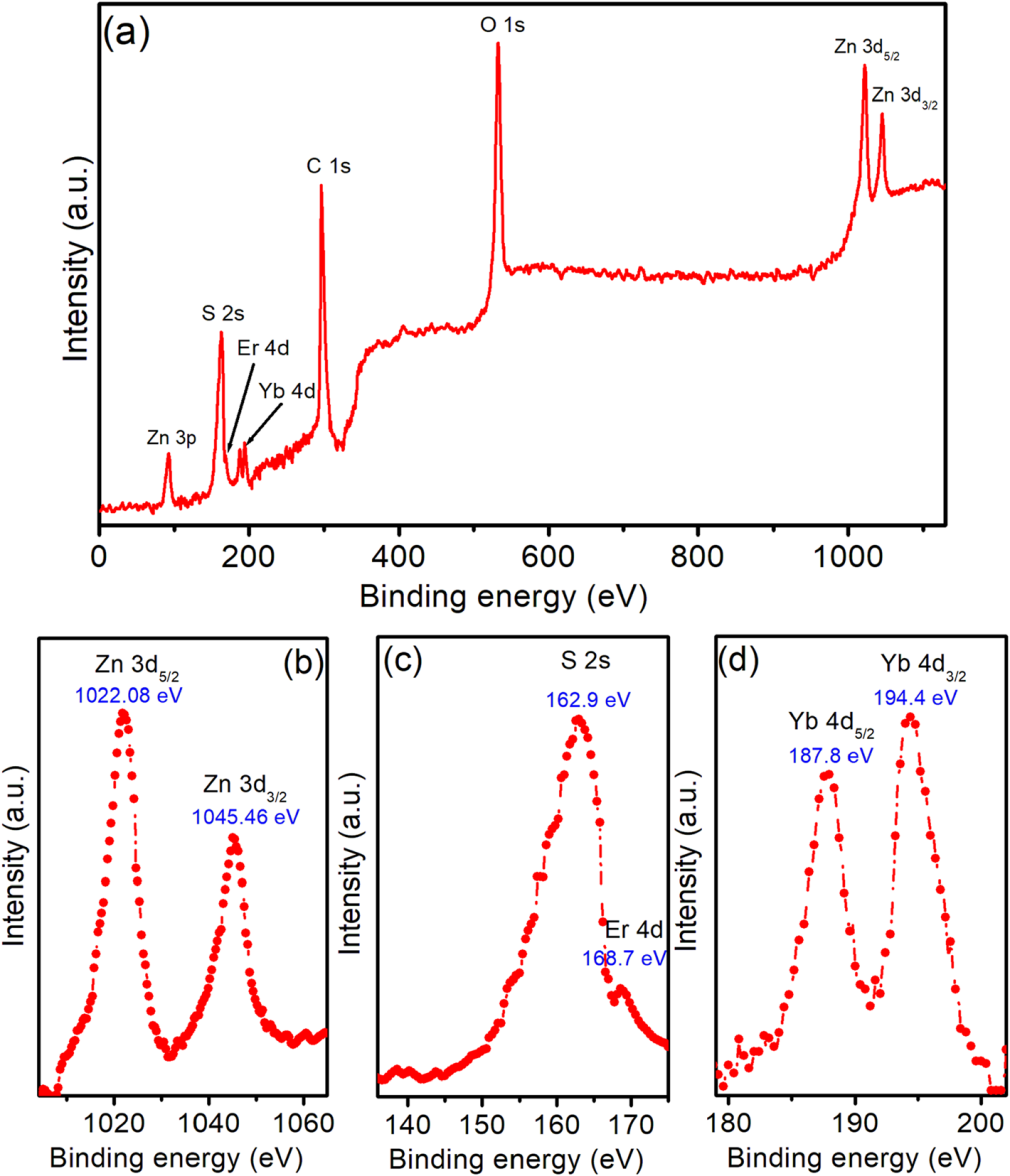
(a) XPS spectrum of ZnEr1%Yb1%S QDs. (b–d) High-resolution XPS spectra of Zn, S, Er, and Yb elements.

XPS spectra of ZnEr1%Yb1%S QDs were investigated to determine the elemental composition as well as the valence states of the doped ions in the crystal lattice. The survey spectra showed the clear presence of peaks characteristic of Zn, S, Er and Yb, confirming the presence of these elements in the material sample (Fig. 3a). The Zn 2p_3/2_ and Zn 2p_1/2_ peaks appeared at 1022.08 eV and 1045.46 eV, respectively (Fig. 3b), consistent with the +2 oxidation state of Zn in the ZnS structure.^29^ Similarly, the S 2s peak was recorded at 162.9 eV (Fig. 3c), characteristic of the S^2−^ anion.^30^ For the rare earth ions, the Er 4d_5/2_ peak was detected at around 168.7 eV (Fig. 3c), confirming the +3 valence state of Er in the crystal lattice.^31^ In particular, the appearance of the Yb 4d_5/2_ and 4d_3/2_ peaks at around 187.8 eV and 194.4 eV (Fig. 3d) indicated the presence of Yb^3+^;^32^ no significant signal was observed in the 181–182 eV region, indicating that Yb exists predominantly in the trivalent state. There were no obvious secondary peaks or shoulders indicating the formation of secondary phases such as isolated rare earth oxides or sulfides, indicating that Er^3+^ and Yb^3+^ were successfully doped into the ZnS lattice without causing phase separation. In addition, the slight shift (∼0.2 eV) of the Zn 2p peak toward higher binding energies compared to pure ZnS suggests a change in the local electron density around the Zn ion, possibly due to electrostatic interactions with the higher-charged rare-earth ions. This result is consistent with the hypothesis that the doping of Er^3+^ and Yb^3+^ resulted in slight distortion of the crystal lattice and induced defect effects, which may influence the optical properties of the material.

**Fig. 4:**
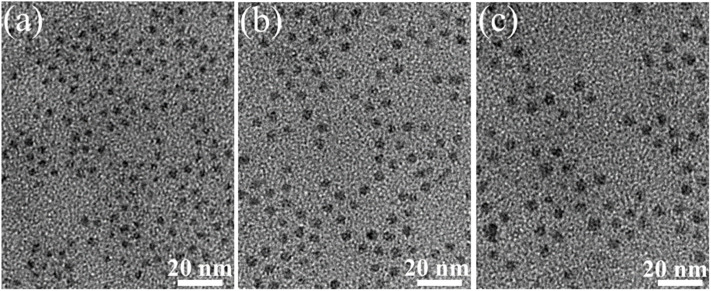
TEM image of the ZnS (a), ZnEr1%Yb0.5%S (b), and ZnEr1%Yb10%S (c) QDs.

Fig. 4 presents the TEM images of ZnS, ZnEr1%Yb0.5%S, and ZnEr1%Yb10%S QDs. The images reveal that the synthesized QDs possess a nearly spherical morphology and are well dispersed without noticeable aggregation. Increasing the Yb^3+^ concentration results in a gradual increase in particle size, while the overall morphology remains unchanged. The average particle sizes of ZnS, ZnEr1%Yb0.5%S, and ZnEr1%Yb10%S QDs were estimated from the TEM analysis to be approximately 6, 7.2, and 8.6 nm, respectively.

### 3.3. Absorption property

**Fig. 5:**
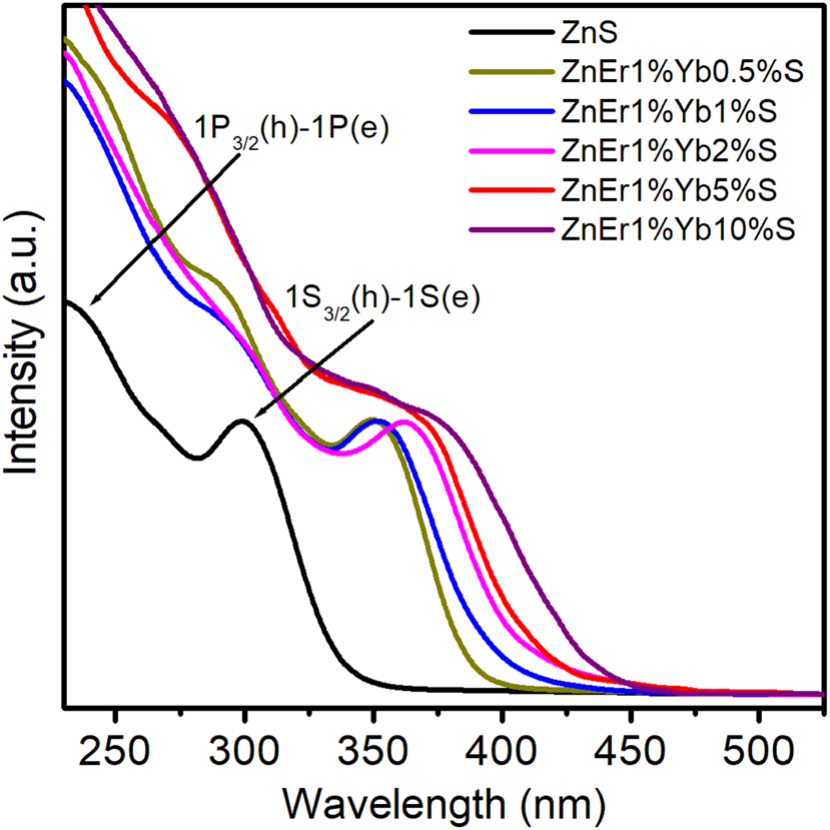
UV-vis spectra of the samples measured at room temperature in hexane, with absorbance values at the absorption peaks below 0.05.

Fig. 5 presents the absorption spectra of pure ZnS and ZnS QDs co-doped with Er^3+^ and Yb^3+^ at different concentrations. For the undoped ZnS QDs (black curve), two characteristic peaks corresponding to the excitonic transitions 1P_3/2_(h)–1P(e) and 1S_3/2_(h)–1S(e) can be clearly identified.^33^ The fundamental excitonic peak 1S_3/2_(h)–1S(e) appears at around ∼300 nm, the energy of this peak is close to the band gap (*E*_g_) of ZnS QDs. Upon co-doping with Er^3+^ and Yb^3+^, these absorption features exhibit a gradual red-shift, while the absorption intensity is also modified. The observed red-shift indicates a slight increase in the average particle size with increasing dopant concentration, consistent with the quantum confinement effect: smaller QDs exhibit a blue-shifted excitonic peak (shorter wavelength), whereas larger QDs show a red-shifted peak (longer wavelength). The incorporation of rare-earth ions may influence crystallization kinetics by reducing surface energy, thereby promoting particle growth. When the Yb^3+^ content increases from 0.5% to 10% (with Er^3+^ fixed at 1%), the 1S_3/2_(h)–1S(e) excitonic peak systematically shifts toward longer wavelengths, demonstrating the size enlargement of Er^3+^/Yb^3+^ co-doped ZnS QDs. Simultaneously, the absorption edge becomes less steep, suggesting a broader size distribution induced by doping. This broadening effect highlights that rare-earth incorporation not only modifies the lattice but also enhances structural inhomogeneity. It is worth noting that samples with higher Yb^3+^ concentrations (5–10%) show more pronounced changes in the absorption edge compared to those with lower concentrations (0.5–1%). This behavior suggests that excessive rare-earth doping may introduce additional defect states or trap levels within the band gap, which act as secondary absorption centers and contribute to the extended absorption tail.

**Fig. 6:**
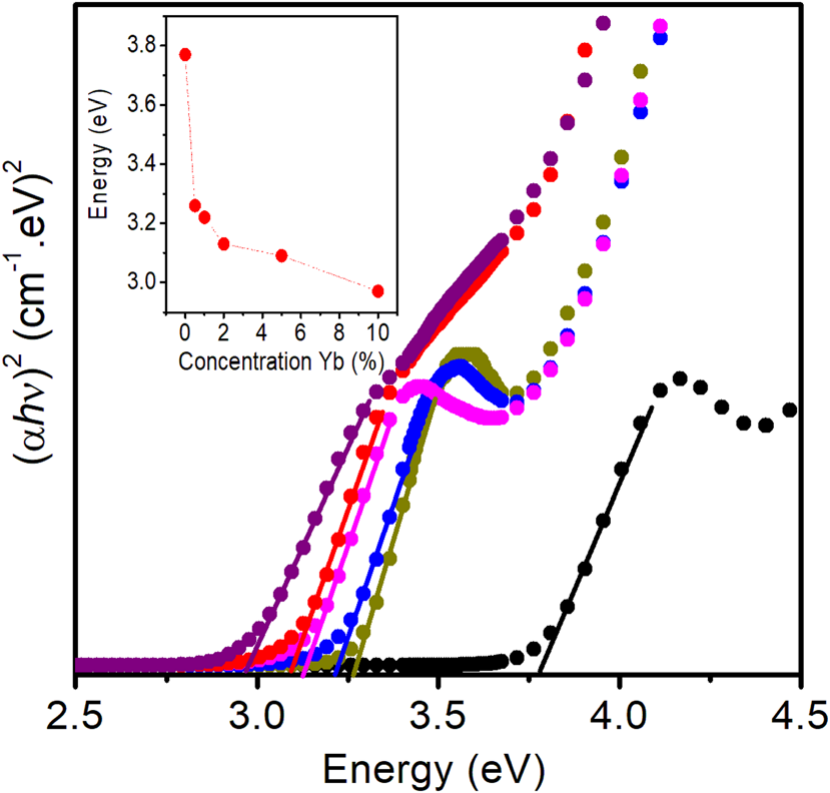
The plot of variation of (*αhν*)^2^*versus* energy (*hν*) (eV) of QDs.

Fig. 6 shows the plot of the dependence of (*αhν*)^2^ on photon energy (*hν*). This is a common method to determine the optical band gap energy (*E*_g_) of QDs, based on the Tauc plot formula:^23,27,34^

5 *αhν* = *A*(*hν* − *E*_g_)^*n*^

In this equation, *A* is a material-dependent constant, *hν* is the photon energy, *α* is the absorption coefficient, and *E*_g_ denotes the optical band gap. The exponent *n* varies with the type of transition, taking values of 2, 3, 1/2, and 1/3 for indirect allowed, indirect forbidden, direct allowed, and direct forbidden transitions, respectively. Since ZnS is a direct band gap semiconductor, *n* = 1/2 is applied.^23,27,34^ Using eqn (5), *E*_g_ of the QDs were determined to be 3.78 eV (ZnS), 3.26 eV (ZnEr1%Yb0.5%S), 3.21 eV (ZnEr1%Yb1%S), 3.12 eV (ZnEr1%Yb2%S), 3.08 eV (ZnEr1%Yb5%S), and 2.96 eV (ZnEr1%Yb10%S), respectively (the inset in Fig. 6).

The size of the ZnS QDs can be determined from the energy of the excitonic peak 1S_3/2_(h)–1S(e) according to the equation:^35^6
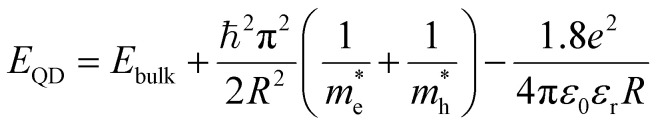
In eqn (6), *E*_QD_ represents the band gap energy of a ZnS QD with radius *R*, while *E*_bulk_ denotes the band gap of bulk ZnS, which is approximately 3.68 eV for the cubic phase. *ħ* corresponds to the reduced Planck's constant. The effective electron mass in ZnS is 
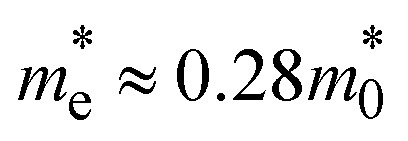
, and the effective hole mass is 
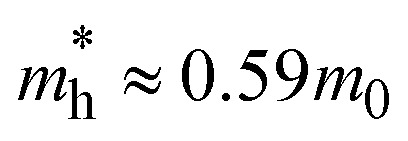
, where *m*_0_ is the rest mass of a free electron. *ε*_0_ stands for the vacuum permittivity, *ε*_r_ is the relative dielectric constant of ZnS (*ε*_r_ ≈ 8.3), and *e* is the elementary charge.^35,36^ Using eqn (6), the estimated mean particle size of the ZnS QDs is about 6.3 nm.

### 3.4. Luminescence properties and upconversion luminescence

**Fig. 7:**
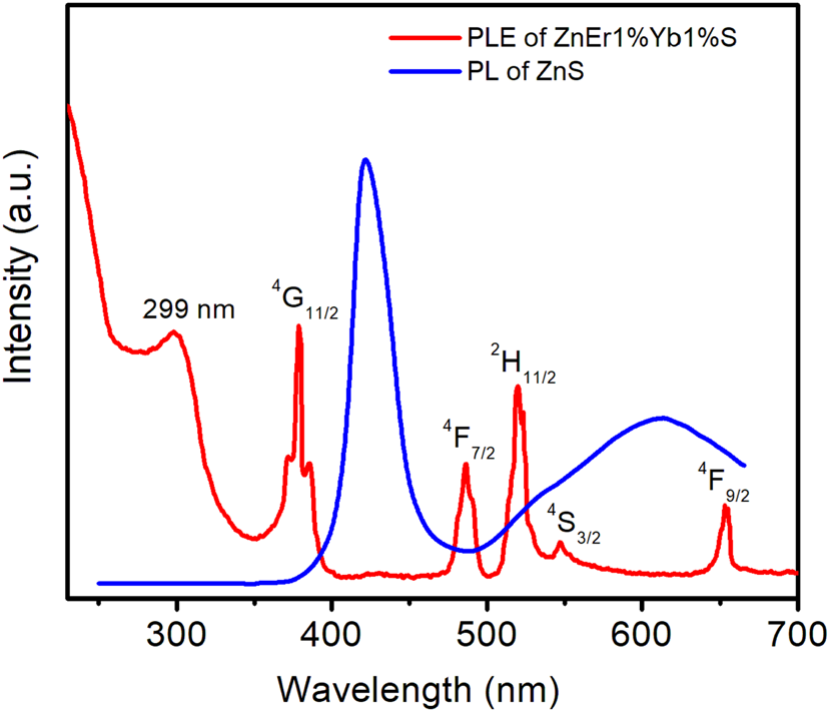
PL of ZnS and PLE of ZnEr1%Yb1%S QDs.

Fig. 7 shows the PL of ZnS (*λ*_ex_ = 225 nm) and PLE of ZnEr1%Yb1%S QDs at fixed emission of 1540 nm (the Er^3+^, ^4^I_13/2_ → ^4^I_15/2_ transition).

The excitation peaks of the PLE spectrum at 378, 489, 520, 549, and 658 nm represent the ^4^I_11/2_ → ^4^G_11/2_, ^4^I_11/2_ → ^4^F_7/2_, ^4^I_11/2_ → ^4^H_11/2_, ^4^I_11/2_ → ^4^S_3/2_, and ^4^I_11/2_ → ^4^F_9/2_ transitions of Er^3+^ ions, respectively.^37^ In addition to the Er^3+^ ion transitions, the PLE spectrum also shows an extended band around 299 nm, which is attributed to the excitation band of the ZnS host. The PLE spectrum in the range of 250–700 nm does not observe the excitation peak of Yb ion because its main absorption peak is located at about 980 nm. Under 225 nm excitation, the PL of ZnS QDs comprises two characteristic peaks: a dominant narrow peak at ≈421 nm and a weaker, broadband emission centered near 613 nm. The 421 nm peak is assigned to near-band-edge (excitonic) recombination of conduction-band electrons with valence-band holes in ZnS.^21,23^ The peak at 613 nm originates from radiative transitions involving surface/defect states within the band gap of the ZnS QDs. The observation results in Fig. 7 show that the PL peak at 421 nm (band-edge exciton recombination of ZnS) does not overlap with the absorption bands (PLE) of Er^3+^, so the Förster-type resonance energy transfer channel from ZnS exciton to Er^3+^ is almost impossible. In contrast, the defect band ∼613 nm of ZnS partially overlaps with the 4f–4f absorption of Er^3+^, allowing energy transfer through trap levels as “sensitizers” for Er^3+^. However, the efficiency of this energy transfer process is not high due to the limited overlap area and the weak oscillator strength of the 4f–4f transitions.

**Fig. 8:**
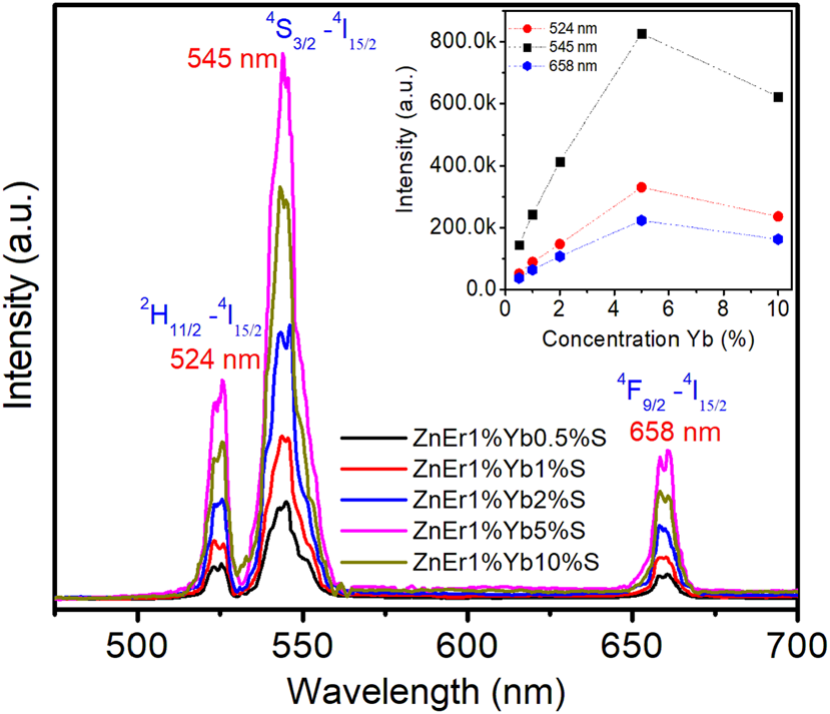
Upconversion PL spectra of QDs with varying Yb concentrations.

Up-conversion (UC) luminescence refers to a nonlinear optical process where a material sequentially absorbs two or more photons of lower energy and then releases a photon of higher energy. This unique emission mechanism has attracted widespread interest because of its versatile potential in diverse areas, including biological imaging, advanced display systems, photovoltaic enhancement, medical diagnostics, and photodynamic therapy. Fig. 8 shows the UC emission spectra of the samples under 980 nm infrared laser excitation at room temperature. The UC photoluminescence features two peaks in the green region and a red peak. Two green emissions centered at ∼524 and ∼545 nm are assigned to the Er^3+^: ^2^H_11/2_–^4^I_15/2_, ^4^S_3/2_–^4^I_15/2_ transitions, respectively.^38,39^ The red band near ∼658 nm originates from the ^4^F_9/2_–^4^I_15/2_ transition. Among the above emission peaks, the peak at 545 nm has a dominant intensity, demonstrating that green emission is the dominant emission of Er^3+^/Yb^3+^ co-doped ZnS QDs.

The intensity difference between the two green peaks (524 and 545 nm) reflects the thermal equilibrium between closely spaced excited states of Er^3+^, enabling the *I*_524_/*I*_545_ ratio to serve as a potential optical thermometer.^40^ Notably, the emission intensity increases significantly as the Yb^3+^ concentration rises from 0.5% to 5%, highlighting the sensitizing role of Yb^3+^ as an efficient energy donor to Er^3+^. However, when the Yb^3+^ content is 10%, the luminescence intensity decreases, indicating the onset of concentration quenching due to Yb^3+^–Yb^3+^ energy migration, Er^3+^ → Yb^3+^ back-transfer, and non-radiative pathways associated with surface defects. In particular, the enhancement of the red 658 nm emission with increasing Yb^3+^ concentration reflects cross-relaxation processes among Er^3+^ levels, suggesting that tuning the Yb^3+^/Er^3+^ ratio not only affects the overall luminescence efficiency but also tailors the emission color balance.

**Fig. 9:**
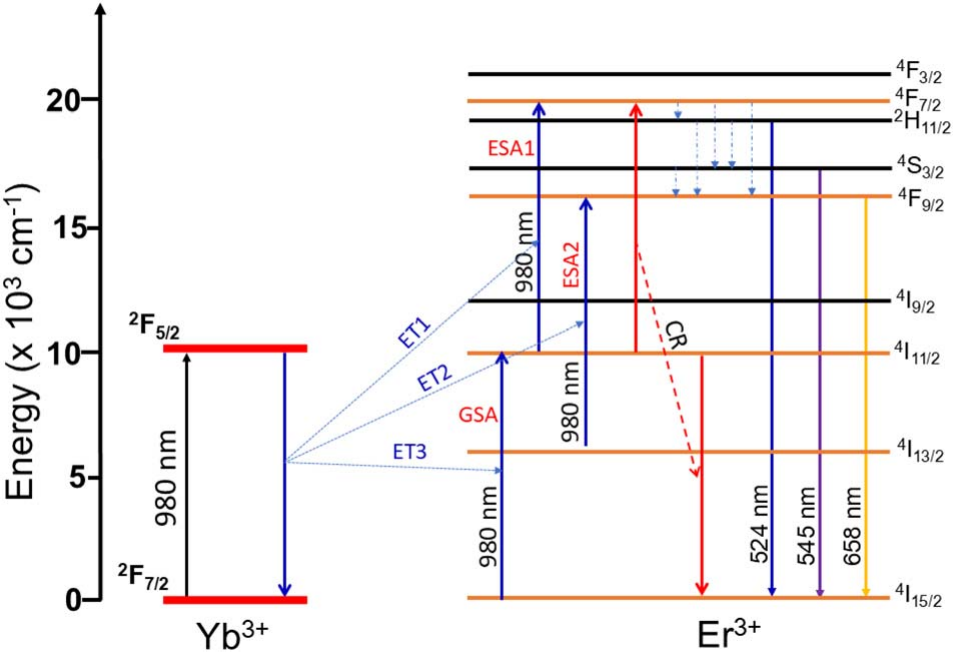
Schematic of UC energy transfer mechanism of Yb^3+^ to Er^3+^ in Er^3+^/Yb^3+^ co-doped ZnS QDs.

The UC luminescence mechanism in Er^3+^/Yb^3+^ co-doped ZnS QDs can be interpreted on the basis of the energy level diagram shown in Fig. 9. Upon excitation with 980 nm radiation, Yb^3+^ ions are promoted from the ground state ^2^F_7/2_ to the excited state ^2^F_5/2_, acting as efficient sensitizers owing to their large absorption cross-section. The stored energy is subsequently transferred to neighboring Er^3+^ ions through successive energy transfer processes (ET1, ET2, ET3).^41^ In the first step (ET1), Er^3+^ is excited from the ground state ^4^I_15/2_ to the ^4^I_11/2_ level, followed by a second transfer (ET2) that promotes Er^3+^ to ^4^F_7/2_. Non-radiative relaxation then populates the thermally coupled ^2^H_11/2_ and ^4^S_3/2_ states, from which radiative transitions to the ground state yield green emissions at 524 nm and 545 nm, respectively. The red emission at 658 nm originates from the ^4^F_9/2_ → ^4^I_15/2_ transition, which can be populated either *via* ET3 from the ^4^I_13/2_ level or through cross-relaxation processes involving two neighboring Er^3+^ ions, where one ion in ^4^S_3/2_ relaxes to ^4^F_9/2_ while transferring energy to another ion. In addition to sensitization by Yb^3+^, Er^3+^ ions can also undergo ground state absorption (GSA) and excited state absorption (ESA1, ESA2) of 980 nm photons, further contributing to the higher-lying states. The UC process is governed by the cooperative interaction between Yb^3+^ and Er^3+^, in which Yb^3+^ serves as an energy donor while Er^3+^ acts as the emitting center, giving rise to the characteristic green and red up-converted emissions. This mechanism highlights the crucial role of energy transfer efficiency and cross-relaxation in determining the intensity ratio and color balance of the luminescence.

**Fig. 10:**
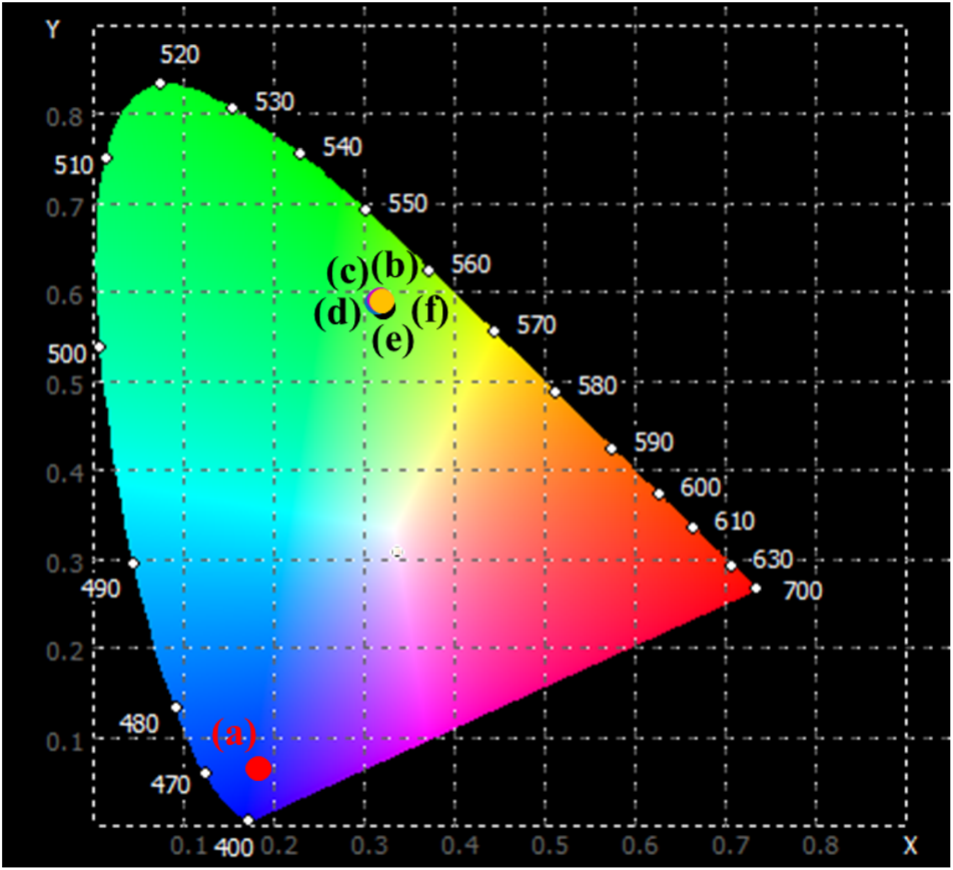
The CIE color coordinates diagram of QDs: ZnS (a), ZnEr1%Yb0.5%S (b), ZnEr1%Yb1%S (c), ZnEr1%Yb2%S (d), ZnEr1%Yb5%S (e) and ZnEr1%Yb10%S (f) with *λ*_exc_ = 980 nm.

The optical emission characteristics of the studied materials were quantitatively analyzed using CIE 1931 chromaticity coordinates, which provide a standardized representation of color based on human visual response. The chromaticity coordinates (*x*, *y*) corresponding to the luminescence of ZnS and Er^3+^/Yb^3+^ co-doped ZnS QDs are represented by the CIE 1931 chromaticity diagram, as illustrated in Fig. 10. The correlated color temperature (CCT) of the samples was estimated using the McCamy empirical relationship:^21,23,42^

7 CCT = −449*n*^3^ + 3525*n*^2^ − 6823*n* + 5520.33

where the parameter *n* is defined by the expression 
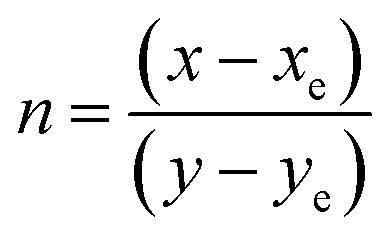
, with the reference chromaticity coordinates *x*_e_ = 0.332 and *y*_e_ = 0.186. The CCT of samples were displayed in Table 2.

**Table 2:** The chromaticity coordinates (*x*, *y*) and the CCT for QDs

Sample	*x*	*y*	CCT (K)
ZnS	0.182	0.067	1621
ZnEr1%Yb0.5%S	0.318	0.588	5050
ZnEr1%Yb1%S	0.312	0.594	4980
ZnEr1%Yb2%S	0.309	0.596	4960
ZnEr1%Yb5%S	0.307	0.598	4940
ZnEr1%Yb10%S	0.308	0.597	4950

The chromaticity coordinates and CCT of the ZnS QDs provide further insight into the influence of Er^3+^/Yb^3+^ co-doping on their emission properties. As summarized in Table 2 and displayed on the CIE 1931 chromaticity diagram (Fig. 10), the pure ZnS sample exhibits coordinates at (0.182, 0.067), which are located in the deep blue region of the spectrum. This position reflects the intrinsic band-edge emission of ZnS QDs. However, upon introducing Er^3+^ and Yb^3+^ ions, the emission coordinates shift dramatically toward the green-yellow region, clustering around (0.31, 0.59). This migration in chromaticity is consistent with the dominance of the characteristic Er^3+^ transitions (^2^H_11/2_/^4^S_3/2_ → ^4^I_15/2_) in the visible domain, facilitated by efficient energy transfer from Yb^3+^ sensitizers. Importantly, the CCT values of the co-doped samples remain relatively stable, ranging narrowly from 4940 to 5050 K, regardless of the Yb^3+^ concentration. Such stability indicates that the emission color of the material is less dependent on the Yb^3+^ impurity concentration, which is very beneficial for potential display and solid-state lighting applications.

From the perspective of perceived color, the pure ZnS QDs exhibits a deep cold-blue emission, consistent with its position in the lower-left region of the CIE diagram. In contrast, all Er^3+^/Yb^3+^ co-doped samples emit in the green-yellow domain, which corresponds to a warm-neutral hue typically associated with CCT values around 5000 K. Such emissions are visually perceived as more balanced and less glaring compared to the harsh bluish output of pure ZnS. The narrow distribution of the chromaticity coordinates further highlights the color uniformity and stability of the doped samples. Therefore, the co-doping process effectively transforms ZnS from a cold-light emitter into a material capable of producing warm-neutral luminescence suitable for lighting and display applications.

**Fig. 11:**
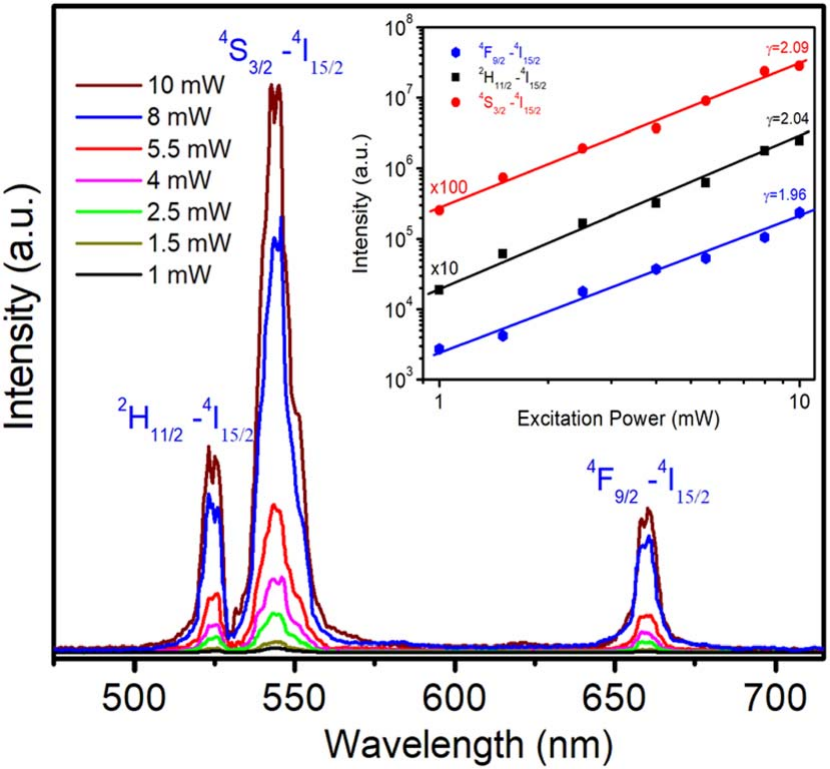
Upconversion emission spectra of ZnEr1%Yb1%S QDs under 980 nm excitation (^4^I_15/2_–^4^I_11/2_) with excitation power varying from 1 to 10 mW. The inset shows the dependence of the upconversion emission intensity on the excitation power.

The UC phenomenon has been explained by several physical mechanisms such as Auger recombination, two-photon absorption, two-step two-photon absorption, and the thermal excitation of surface states.^1–5^ To gain a deeper understanding of the UC mechanism, we investigated the power-dependent luminescence. Fig. 11 displays the UC emission spectra of ZnEr1%Yb1%S QDs when excited at 980 nm (^4^I_15/2_–^4^I_11/2_) with varying excitation power from 1 to 10 mW. The observation results from Fig. 11 show that when the excitation power increases from 1 to 10 mW, the intensity of the emission peaks of Er^3+^ ion at 524, 545, and 658 nm all increase.

The quantitative correlation between the UC emission intensity and the incident excitation power can be expressed by a power-law dependence, which is generally formulated as:^4,5,43^

8 *I*_UC_ ∼ *P*^*n*^

In this relation, *I*_UC_ denotes the UC luminescence intensity associated with the Er^3+^ ions, *P* represents the incident pump power, and *n* corresponds to the number of photons involved in the excitation process. The value of *n* depends on the observed UC luminescence mechanism. When *n* ≈ 2, the results confirm that the dominant UC mechanism is a two-photon process, in which Er^3+^ ions absorb two sequential photons (either directly or *via* energy transfer) from sensitizer Yb^3+^ ions. In case *n* ≈ 3, the UC mechanism requires the absorption of three photons. Deviations below the ideal value of 2 (*e.g.*, *n* ∼ 1.7–1.8) imply that although the process is fundamentally two-photon, additional non-radiative channels, such as multiphonon relaxation, cross-relaxation, or back energy transfer, reduce the effective photon number. Conversely, values of *n* > 2 reflect the involvement of higher order processes or amplification-like mechanisms, where more photons are required to initiate efficient UC emission.

To evaluate this parameter experimentally, the logarithmic plots of *I*_UC_ as a function of *P*^*n*^ were constructed for the characteristic emission transitions, as displayed in the inset in Fig. 11. The obtained data show good linearity between *I*_UC_ and *P*^*n*^. From the linear fits, the extracted slope values were found to be 1.96, 2.04, and 2.09 for the ^4^F_9/2_ → ^4^I_15/2_, ^2^H_11/2_ → ^4^I_15/2_, and ^4^S_3/2_ → ^4^I_15/2_ transitions, respectively. These values, which are close to the theoretical value of 2, clearly demonstrate that the green and red UC emissions in Er^3+^/Yb^3+^ co-doped ZnS QDs are governed predominantly by a two-photon absorption mechanism. Moreover, the slight deviations from the ideal integer value indicate that additional non-radiative processes, such as energy transfer, multiphonon relaxation, or cross-relaxation, may also contribute to the population dynamics of these excited states.

The two-photon absorption mechanism with UC luminescence of Er^3+^/Yb^3+^ co-doped ZnS QDs is observed in Fig. 9. In the Er^3+^/Yb^3+^ co-doped ZnS QDs, the UC luminescence is governed by a two-photon absorption process through sequential energy transfer. The Er^3+^ ions in the ground state absorb photons with wavelength 980 nm and move to the ^4^I_11/2_ level. Subsequently, energy migration among Er^3+^ ions takes place, which can be expressed by the following equation:

9 ^4^I_11/2_ + ^4^I_11/2_ → ^4^I_15/2_ + ^4^F_7/2_

Under near-infrared excitation (980 nm), Yb^3+^ ions with a large absorption cross-section are excited from the ^2^F_7/2_ ground state to the ^2^F_5/2_ excited state. The stored energy is then transferred to Er^3+^ ions, promoting them from the ^4^I_15/2_ ground state to the ^4^I_11/2_ intermediate level. The absorption of a second photon then promotes the ion from the ^4^I_11/2_ state to the ^4^F_7/2_ state. Radiative relaxation from these states to lower-lying levels results in characteristic visible emissions: green (524, 545 nm) and red (658 nm). Thus, two near-infrared photons are effectively converted into one higher-energy photon, producing the UC phenomenon. The ZnS host, with its wide bandgap and quantum confinement effect, not only stabilizes the dopant ions but also suppresses non-radiative pathways, thereby enhancing the overall emission efficiency of the material system.

**Fig. 12:**
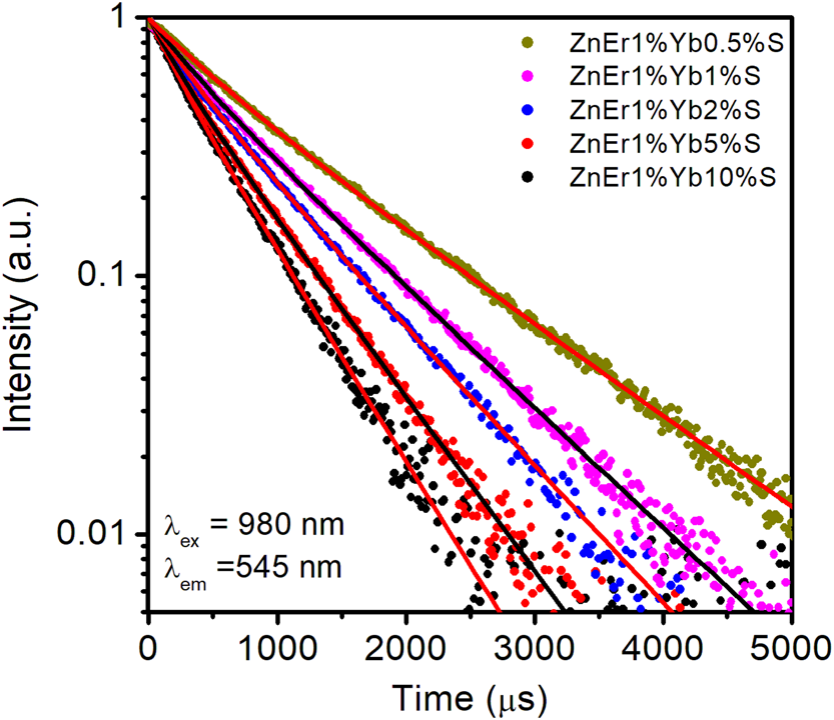
Decay time curves of ZnEr1%Yb*x*%S QDs with varying Yb concentrations.

To gain deeper insight into the energy transfer mechanism between Er^3+^ and Yb^3+^ ions, the luminescence decay curves of ZnEr^3+^1%Yb^3+^*x*S (*x* = 0.5–10%) QDs were systematically recorded under 980 nm excitation (Fig. 12). The decay signals were monitored at 545 nm peak, which corresponds to the characteristic ^4^S_3/2_ → ^4^I_15/2_ transition of Er^3+^ ions. This emission band has the greatest intensity and is particularly sensitive to energy transfer processes, thereby serving as a reliable probe for evaluating the efficiency of Yb^3+^ → Er^3+^ energy transfer.

The PL decay curves were evaluated by applying a bi-exponential fitting model, as expressed in equation:^21,23,44,45^10
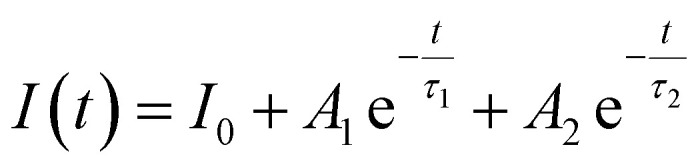
In this formulation, *I*(*t*) denotes the PL intensity at a given time *t*, while *I*_0_ corresponds to the initial emission intensity at *t* = 0. The coefficients *A*_1_ and *A*_2_ represent the relative amplitudes of the fast and slow decay processes, associated with the lifetimes *τ*_1_ and *τ*_2_, respectively. The average lifetime 〈*τ*〉 is given by the following equation:^21,23^11
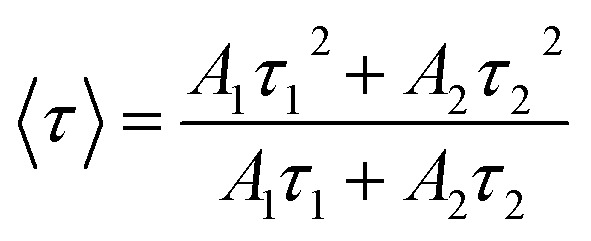


The experimental decay curves exhibit non-single-exponential behavior, reflecting the coexistence of multiple de-excitation pathways involving both radiative and non-radiative processes. Therefore, the decay kinetics were fitted using a bi-exponential function (eqn (10)), which provides a more accurate description by accounting for the presence of fast and slow decay components. The fast component is generally associated with efficient energy transfer and cross-relaxation processes, while the slower component is attributed to the intrinsic radiative lifetime of Er^3+^ ions in the ZnS host lattice. The values of *A*_*i*_, *τ*_*i*_, and the average lifetime 〈*τ*〉 obtained from the fitting procedure are presented in Table 3.

**Table 3:** *τ*_*i*_ and *A*_*i*_ values for ZnS:Er^3+^/Yb^3+^ samples with all doping ratios by monitoring 545 nm

Sample	*A*_1_	*τ*_1_ (µs)	*A*_2_	*τ*_2_ (µs)	*τ*_ave._ (ms)
ZnEr1%Yb0.5%S	0.35	0.24	0.65	1.05	0.96
ZnEr1%Yb1%S	0.33	0.2	0.67	0.82	0.75
ZnEr1%Yb2%S	0.28	0.19	0.72	0.68	0.63
ZnEr1%Yb5%S	0.31	0.16	0.69	0.57	0.52
ZnEr1%Yb10%S	0.34	0.12	0.66	0.49	0.45

As shown in Table 3, the average lifetime 〈*τ*〉 of the Er^3+^ emission at 545 nm systematically decreases with increasing Yb^3+^ concentration. Specifically, 〈*τ*〉 reduces from 0.96 ms for the ZnS:Er1%Yb0.5%S sample to only 0.45 ms for ZnS:Er1%Yb10%S. This reduction in lifetime indicates that higher concentrations of Yb^3+^ ions significantly enhance the probability of energy transfer processes. Under 980 nm excitation, Yb^3+^ ions act as sensitizers by efficiently absorbing incident photons and subsequently transferring the energy non-radiatively to neighboring Er^3+^ ions. At low Yb^3+^ content, the number of available sensitizers is limited, resulting in weaker energy transfer and thus longer observed 〈*τ*〉 values. Conversely, as the Yb^3+^ content increases, the sensitive ion density surrounding each Er^3+^ ion becomes larger, thus facilitating the Yb^3+^ → Er^3+^ energy transfer process to occur more frequently.^46^ This enhanced transfer efficiency accelerates the depopulation of the Er^3+^ excited state, manifested as a reduced average lifetime.^47^ The presence of both fast and slow components in the decay fitting further confirms the coexistence of rapid non-radiative energy transfer and the intrinsic radiative relaxation of Er^3+^. The progressive quenching of 〈*τ*〉 with increasing Yb^3+^ therefore provides compelling evidence that the UC process in Er^3+^/Yb^3+^ co-doped ZnS QDs is predominantly governed by an Yb^3+^-sensitized energy transfer mechanism. Moreover, the results suggest an optimal doping threshold: while higher Yb^3+^ concentrations promote stronger sensitization, excessive doping can also introduce concentration quenching due to cross-relaxation and energy migration among Yb^3+^ ions, which may reduce overall luminescence efficiency.

## 4. Conclusion

In summary, Er^3+^/Yb^3+^ co-doped ZnS QDs with cubic structure were successfully synthesized by a wet-chemical synthesis route. XRD results confirmed lattice expansion as the (111) diffraction peak shifted from 28.423° (pure ZnS) to 28.062° (ZnEr1%Yb10%S), corresponding to an increase in lattice parameter from 5.412 Å to 5.483 Å. TEM analysis revealed nearly spherical particles with sizes ranging from 6 to 8.6 nm, while XPS confirmed the +3 oxidation states of Er and Yb ions. Optical absorption spectra exhibited a progressive bandgap narrowing from 3.78 eV (ZnS) to 2.96 eV (ZnEr1%Yb10%S), indicating size enlargement and defect-related transitions. Photoluminescence showed two distinct emissions: a near-band-edge peak at 421 nm and a defect-related band at 613 nm. More importantly, under 980 nm excitation, UC spectra revealed two green peaks (524, 545 nm) and a red peak (658 nm), with maximum intensity achieved at 5% Yb^3+^ doping. Beyond this concentration, quenching occurred due to Yb^3+^–Yb^3+^ migration and Er^3+^ → Yb^3+^ back-transfer. Power-dependent studies yielded slope values of ∼2, confirming a two-photon mechanism, while lifetime decay curves showed systematic reduction of average *τ* from 0.96 ms (0.5% Yb) to 0.45 ms (10% Yb), evidencing enhanced Yb^3+^ → Er^3+^ energy transfer. Chromaticity coordinates shifted from (0.182, 0.067) for ZnS to ∼(0.31, 0.59) for co-doped samples, with stable CCT around 4940–5050 K, corresponding to warm green-yellow emission. These detailed findings provide clear mechanistic understanding of the role of Er/Yb doping in tuning structural and UC luminescence properties of ZnS QDs, paving the way for their applications in optical thermometry, bioimaging, display, and solid-state lighting technologies.

## Conflicts of interest

There are no conflicts to declare.

## Data Availability

The data supporting this study's findings are available on request from the corresponding author, [Nguyen Xuan Ca, E-mail: canx@tnus.edu.vn]. Data are not publicly available for privacy reasons.
